# From habits to harm: the effects of lifestyle, sleep, and fitness on injury risk in students using a structural equation modeling approach

**DOI:** 10.3389/fpubh.2025.1664822

**Published:** 2025-11-18

**Authors:** Mario Kasović, Bruno Škrinjarić, Tomáš Vespalec, Milan Mojžíš, Damir Knjaz

**Affiliations:** 1 Department of General and Applied Kinesiology, Faculty of Kinesiology, University of Zagreb, Zagreb, Croatia; 2 Department of Physical Activities and Health Sciences, Faculty of Sports Studies, Masaryk University, Brno, Czechia; 3 Institute of Economics Zagreb, Zagreb, Croatia; 4 Department of Kinesiology of Sports, Faculty of Kinesiology, University of Zagreb, Zagreb, Croatia

**Keywords:** lifestyle habits, physical fitness, sleep quality, sports activity, injury occurrence

## Abstract

**Background:**

University students often deal with a mix of challenges — from frequent sports and recreational injuries to habits like smoking, drinking alcohol, or consuming energy drinks, along with irregular sleep and different levels of physical activity. This study examines the links between lifestyle habits, sports participation, sleep quality, self-perceived fitness, and the likelihood of getting injured.

**Methods:**

In a cross-sectional survey of 933 first-year students (mean age 20.1 ± 2.5), participants reported lifestyle indicators (cigarettes, alcoholic drinks, energy drinks), sports activity (training volume, athlete/competitive status, sport diversity), sleep quality, self-rated fitness, and the number of injuries causing class absence. Partial least squares structural equation modeling evaluated the measurement properties and the structural paths.

**Results:**

Sports activity had a strong, positive association with perceived physical fitness. The interaction of lifestyle and sports activity is negatively associated with fitness, indicating that unhealthy habits attenuate sport-related fitness gains. Lifestyle was negatively associated with sleep quality, while better sleep quality positively predicted fitness. Physical fitness did not show a significant direct association with injury counts. Poorer sleep quality was associated with a higher number of injuries.

**Conclusion:**

In this large student sample, sleep quality emerged as a pivotal, modifiable protector—linked positively to fitness and negatively to injury risk—whereas sports participation improved fitness, but its benefits were undermined by unhealthy lifestyle behaviours. Injury prevention efforts in student populations should therefore combine promotion of healthy lifestyle choices, sleep hygiene interventions, and safe sport practices.

## 1 Introduction

In today’s dynamic and technology-driven society, characterized by rapid lifestyle changes and increasing demands on individuals, understanding the complex interactions between lifestyle, health, and safety has become essential for promoting overall well-being and preventing disease (1). Of particular importance is examining how everyday behavioral factors such as physical activity, nutrition, sleep, and social engagement affect injury development and mental health among young people, especially university students (2–4). Injuries represent a major global public health concern with considerable social and economic consequences, influencing not only physical health but also social participation, educational attainment, and work capacity (5, 6). Statistical trends indicate a continuous increase in injury rates among young and physically active populations, highlighting the need for high-quality research to identify causes and improve prevention (1, 5).

Among students, injuries most frequently occur during sports participation, recreational activities, or within student facilities, leading to interruptions in academic performance and increased healthcare expenditures (7–10). Understanding the causes and preventive factors of such injuries is therefore crucial for reducing their prevalence and enhancing both safety and quality of life in student populations. Furthermore, a growing number of students report difficulties with sleep, stress, and irregular daily routines, all of which further deteriorate health status and elevate injury risk (11–14). The central challenge lies in connecting these interrelated factors and developing effective strategies to promote a healthier and safer university environment.

The main research question addressed in this study is: *What are the key factors associated with injury occurrence, and what are the causal relationships among these factors?* In addition, the study investigates how individual differences such as gender, age, and social background affect both injury incidence and sleep problems. Special attention is given to the roles of physical activity, sports participation, stress exposure, and lifestyle habits in shaping injury risk, and to identifying how these elements can inform effective preventive strategies. The research further explores associations between sleep quality and injury frequency, as well as the combined impact of stress and lifestyle on overall student health. Ultimately, the study aims to identify the most significant predictors and risk factors, thereby providing a foundation for evidence-based preventive and educational programs designed to enhance safety, health, and well-being among students. Moreover, it seeks to clarify how positive changes in lifestyle habits can contribute to reducing injury occurrence and improving overall quality of life.

This research contributes to understanding the complex interrelations between lifestyle, injuries, and sleep quality among university students. It presents a comprehensive dataset illustrating how sports participation and stress influence injury occurrence and how these factors are connected to sleep quality. Moreover, the study underscores the importance of individualized preventive strategies to reduce injury risk, particularly within students’ sports and recreational contexts. The findings further highlight the need to promote a holistic approach to healthy living—emphasizing education on sleep quality, stress management, and appropriate physical activity choices. More broadly, the results provide valuable insights for developing guidelines and policies aimed at enhancing safety and health among young people, thereby informing future preventive programs within university settings and healthcare institutions.

## 2 Literature review and hypotheses

Recent research reframes injuries not as random events but as outcomes of complex interactions among social, psychological, and physiological factors, where both internal and external determinants create predictable, modifiable risk (5, 15–17). In university populations, sports and recreational activities are consistently identified as prominent contexts for injury occurrence, and injuries among students commonly interfere with academic obligations and increase demands on health services (7–10). Studies across different settings further highlight that injury risk is multifactorial, with behavioral factors (e.g., alcohol use), demographic characteristics, and activity type shaping patterns of harm (4, 18–21). Our research supports a preventive perspective that targets context-specific and individual risk factors rather than treating injuries as isolated or unavoidable incidents (15, 16).

Lifestyle is a multidimensional construct that integrates dietary choices, physical activity, substance use, social behaviors, and daily routines, each interacting with environmental and personal factors to shape health trajectories (22, 23). Regular sport participation and higher activity volumes are robustly associated with better physical fitness and mental well-being, and they promote cognitive and psychosocial benefits that can support academic performance (24–27). Mechanistic research further suggests exercise-related neuroplastic and epigenetic changes that underpin cognitive and physiological improvements (28, 29). Nevertheless, unhealthy behaviors such as tobacco, excessive alcohol, or frequent energy-drink use can attenuate the positive effects of sport and reduce recovery capacity, indicating that the benefits of sports participation are contingent on broader lifestyle patterns (30–34). Thus, an integrative view that considers both sport engagement and co-occurring lifestyle behaviors is necessary when assessing fitness and its health implications in student populations (35, 36).

Sleep quality is tightly interwoven with lifestyle behaviors: diet, substance use, physical activity, and stress levels all influence sleep patterns, which in turn affect cognition, mood, and physical recovery (13, 37, 38). In adolescents and young adults, inadequate or disturbed sleep is linked to impaired concentration, mood disturbances, and poorer academic functioning (11, 14, 39). Physical activity typically supports better sleep, whereas stimulant use and high stress undermine sleep continuity and restorative processes (13, 25, 27). Given the frequent sleep perturbations reported by students and their associations with lifestyle and stress, sleep quality is plausibly both an outcome of lifestyle and an upstream determinant of health and performance (40, 41).

Emerging evidence positions sleep quality as a key factor in injury risk and recovery. Poor or insufficient sleep impairs neuromuscular control, attention, and reaction time, thereby increasing vulnerability to musculoskeletal and other injuries; conversely, adequate sleep supports recovery and performance (3, 42–44). Occupational and athletic studies indicate that sleep disturbances and daytime sleepiness are associated with elevated injury risk, suggesting that sleep operates as a modifiable mediator between lifestyle, fitness, and harm (44, 45). For student populations where sport participation, irregular schedules, and stress commonly co-occur, integrating sleep into preventive strategies may therefore yield important benefits for both health and academic continuity (46).

Taken together, the literature supports an integrative, preventive framework in which lifestyle behaviors, sport participation, and sleep quality interact to shape physical fitness and injury risk. Building on this synthesis, we test the following hypotheses:

*H1:* Lifestyle (LFS) has a statistically significant association with the level of fitness (FIT), with students leading more active and healthier lifestyles demonstrating higher levels of physical conditioning, better bodily functionality, and overall well-being compared to those with less active and unhealthier lifestyles.

*H2:* Engagement in sports (SPO) has a significant positive direct correlation with the level of fitness (FIT) (H2a), but also jointly with lifestyle (LFS) contributes to the improvement of fitness (FIT) (H2b). It is expected that students who regularly participate in sports activities will show higher levels of physical fitness, healthier lifestyles, and better overall well-being compared to those who are inactive or participate in sports less frequently.

*H3:* Lifestyle (LFS) has a statistically significant and positive association with sleep quality (SLQ), with students leading more active, healthier, and balanced lifestyles achieving better sleep quality.

*H4:* The level of sleep quality (SLQ) is statistically significantly and positively correlated with the level of fitness (FIT), whereby better sleep quality contributes to higher physical conditioning and overall well-being, while poor sleep quality negatively affects the ability to maintain optimal fitness.

*H5:* A higher level of fitness (FIT) is positively associated with the reduction of injuries (INJ), with students who have better physical conditioning having a lower likelihood of experiencing injuries related to sports or other physical activities.

*H6:* A higher level of sleep quality (SLQ) is statistically significantly and positively correlated with the number of injuries (INJ), where a poorer perception of sleep quality is associated with a higher number of injuries, while better sleep quality can reduce the risk of injuries.

**Figure 1:**
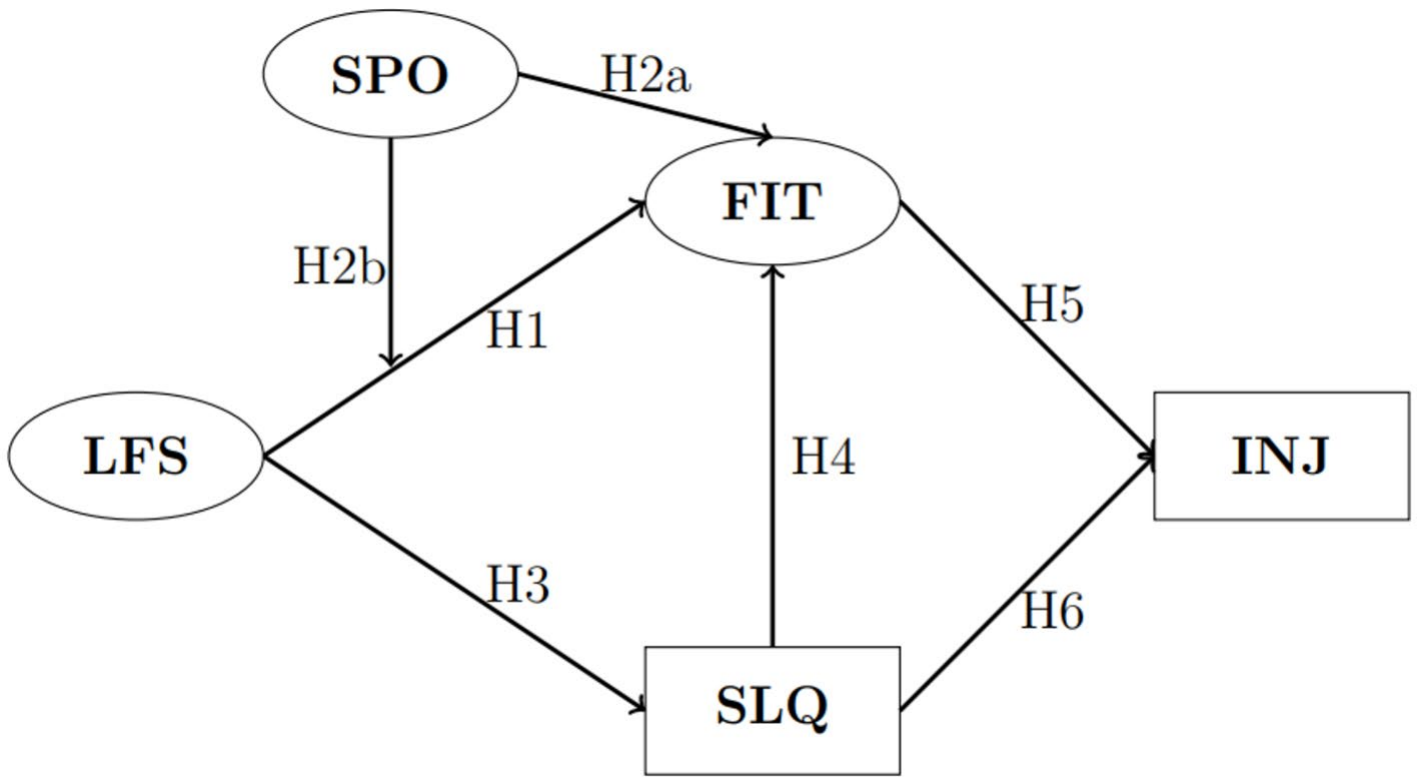
Research model. Circles represent latent (multi-item) estimated variables, and rectangles represent single-item variables. LFS, lifestyle; SPO, sport; FIT, fitness; SLQ, sleeping quality; INJ, number of injuries.

Our empirical model is presented in Figure 1.

## 3 Methodology

### 3.1 Data collection and sample

The measurement was conducted at the beginning of the second semester of the academic year between 2023 and 2025 at the Faculty of Kinesiology, University of Zagreb. A total of 933 first-year students participated in the study. Participation in the study was completely voluntary, and students were informed about the purpose and methodology of the research before completing the questionnaire. Data were collected via an online platform that ensured easy access and anonymity for participants, thus ensuring trust and the protection of participants’ privacy.

Table 1 provides a comprehensive overview of the demographic, socioeconomic, and health-related characteristics of the 933 respondents included in the study. Regarding gender distribution, males constituted a larger proportion of the sample (*n* = 587; 62.9%), while females represented 37.1% (*n* = 346). Most participants (65.6%, *n* = 612) resided in urban areas with populations exceeding 10,000, whereas 34.4% (*n* = 321) lived in rural communities, suggesting that the sample was more urban-centric. Health status was predominantly positive, with a combined 85.8% of respondents rating their health as either “very good” (*n* = 512; 54.9%) or “excellent” (*n* = 288; 30.9%). A smaller segment rated their health as merely “good” (*n* = 124; 13.3%), and only 1.0% (*n* = 9) described their health as “bad,” indicating generally high self-perceived health among the participants.

**Table 1:** Sample characteristics.

Respondent characteristics	*n*	%
*Gender*		
Female	346	37.1%
Male	587	62.9%
*Residence status*		
Rural (<10,000 residents)	321	34.4%
Urban (>10,000 residents)	612	65.6%
*Health status*		
Bad	9	1.0%
Good	124	13.3%
Very good	512	54.9%
Excellent	288	30.9%
*Material status*		
Below average	18	1.9%
Average	769	82.4%
Above average	146	15.6%
*Athlete status*		
Non athlete	218	23.4%
Athlete	715	76.6%
*Injury status*		
Not injury	373	40.2%
Injury	554	59.8%

	Mean (Median)	S.d.
Respondents’ age	20.1 (20)	2.5
Respondents’ height (cm)	177.1 (178)	9.3
Respondents’ weight (kg)	72.9 (73)	12.2

“S.d.”, stands for standard deviation.

Material status, used as a proxy for socioeconomic standing, was overwhelmingly rated as “average” by respondents (*n* = 769; 82.4%), with fewer individuals identifying as either “below average” (*n* = 18; 1.9%) or “above average” (*n* = 146; 15.6%). Most of the sample (*n* = 715; 76.6%) identified as athletes, suggesting a strong representation of physically active individuals. In contrast, non-athletes comprised 23.4% (*n* = 218) of the cohort. Notably, 59.8% (*n* = 554) of participants reported having sustained at least one injury, compared to 40.2% (*n* = 373) who had not, which may be related to the high proportion of athletes in the sample.

Regarding anthropometric and age-related variables, the mean age of respondents was 20.1 years (SD = 2.5), with a median age of 20, indicating a relatively young sample population. The average height was 177.1 cm (SD = 9.3), and the average weight was 72.9 kg (SD = 12.2), consistent with normative values for young adults. These characteristics provide important context for interpreting subsequent analyses and suggest that the sample is relatively healthy, young, and physically active, with a moderate socioeconomic background and a predominance of urban dwellers.

### 3.2 Variables

*Injuries (INJ)* represent the numerical count of injuries that have caused absenteeism from classes and difficulties in performing academic duties (19). The number of injuries among students is a significant factor affecting their academic life, as well as their overall well-being (7, 8).

*Sleep quality (SLQ)* was measured utilizing an online questionnaire based on the Pittsburgh Sleep Quality Index (PSQI) developed by Buysse et al. (47) The questionnaire included a series of questions aimed at the subjective evaluation of various aspects of sleep to gain a comprehensive insight into sleep patterns and possible sleep disturbances among students. The Pittsburgh Index comprises a questionnaire with 19 questions. The components are rated on a scale from 0 to 3, where a lower score indicates the absence of problems, while a higher score indicates deterioration. By combining certain questions, seven main components are formed: (1) subjective sleep quality, (2) sleep latency, (3) sleep duration, (4) sleep efficiency, (5) sleep disturbances, (6) use of sleeping medication, and (7) daytime dysfunction. The sum of all components forms a scale ranging from 0 to 21 points. PSQI entered our model in its inverse form, meaning that a lower score indicates poorer sleep quality, while a higher score indicates greater sleep quality.

The level of perceived physical *fitness (FIT)* was assessed with the following set of questions: (FIT1) “How would you rate your health status?,” (FIT2) “How would you rate your life quality status?,” (FIT3) “How would you rate your physical fitness?,” (FIT4) “In the last 30 days, how many hours per day have you engaged in high physical activity?,” and (FIT5) “In the last 30 days, how many hours per day have you engaged in light physical activity?.” The first question FIT1 assesses the general health status of each participant. It considers self-assessment of health, which may include the frequency of illnesses, the presence of chronic conditions, or the general feeling of physical well-being. Health status is crucial for understanding overall readiness and capacity to engage in physical activities (48). The second question FIT2 captures participants’ perception of their quality of life, including satisfaction with personal, social, and professional aspects of life. Quality of life influences motivation, mental health, and the degree of engagement in physical activities (49). The third question FIT3 evaluates participants’ overall physical readiness, including assessments of strength, endurance, flexibility, and agility. Fitness is key to the ability of participants to actively engage in various physical activities. The fourth question FIT4 records the average number of hours per day that participants dedicate to vigorous physical activity over the last 30 days. Vigorous activities include exercises that significantly increase heart rate and energy expenditure. The final question tracks the average number of hours per day spent on light physical activities. Such activities include daily walks and other forms of movement that keep the body active without causing significant physical exertion. Analyzing these variables enables a comprehensive review of how physical activity and general living conditions affect participants’ physical fitness, providing relevant data for further conclusions and recommendations. Responses ranged from 1 (very poor fitness) to 10 (excellent fitness) (50). Research has shown that this assessment is significantly associated with objectively measured physical fitness and perceived well-being (51, 52).

*Lifestyle (LFS)* encompasses various aspects of participants’ behavior that can affect their health outcomes and fitness levels. The following items were analyzed: (LFS1) “Respondents’ number of cigarettes daily”, (LFS2) “Respondents’ number of drinks during a night out”, and (LFS3) “Respondents’ number of energy drinks daily”. The first item, LFS1, measures the average number of cigarettes each participant smokes daily. Smoking is a known risk factor for various health issues, including reduced cardiovascular fitness and respiratory function, and can negatively impact overall fitness (32). The second item (LFS2) refers to the average number of alcoholic beverages a participant consumes during a night out (53, 54). Excessive alcohol consumption can have negative health consequences, including an increased risk of injury and decreased physical abilities, which can affect overall fitness levels. The final item, LFS3, records the average number of energy drinks each participant consumes daily (31). Regular consumption of energy drinks can lead to sleep disturbances and cause cardiac issues, negatively affecting physical fitness and overall health. The purpose of analyzing these variables is to gain a better understanding of how different aspects of lifestyle contribute to participants’ overall fitness level and health (30).

The relationship between sports activities and injuries represents an important aspect of students’ physical health and can significantly impact their academic and personal well-being. Although sport and physical activity offer many benefits, including improved physical fitness, social skills, and mental health, they also carry certain risks of injury (55, 56). In this study, students’ *sports activity (SPO)* includes the following items: (SPO1) the diversity or number of different sports they participate in, (SPO2) their athlete status, (SPO3) weekly training volume, and (SPO4) competitive sport status. The first item, SPO1, provides insight into the variety of sports activities and offers answers regarding which activities pose higher risks for students. The second item, SPO2, indicates whether the student is actively engaged in sports, and fourth item SPO4 specifies the level at which the student participates in sports—ranging from recreational activities for enjoyment (Recreational level), students who take sports seriously by participating in local leagues or competitions (Competitive level), and those who compete at the highest level as members of sports teams or clubs (Professional level) (57). It is assumed that each level has its own specificities and presents different risks for students. Finally, the third item, SPO3, describes the numerical value of the total hours spent in sports during a week (58, 59).

Table 2 presents the descriptive statistics of all observed variables forming the latent constructs used in the model. The mean PSQI-based sleep quality score (after inversion for interpretability) indicates generally good sleep among respondents (mean = 16.7 ± 1.9), while the average number of injuries reported during the academic year was relatively low (mean = 1.1 ± 1.3). Lifestyle indicators show limited smoking (mean = 1.2 cigarettes/day) and moderate alcohol consumption during nights out (mean = 3.2 drinks), with low energy-drink intake (mean = 0.9 per day). Sport-related variables reveal high engagement in physical activity—students trained on average 7.5 h per week, most were active athletes (mean = 0.8 ± 0.4), and two-thirds competed at least at the local level (mean = 1.7 ± 0.6). Fitness-related indicators suggest overall favorable self-rated health (mean = 3.2 ± 0.7) and life quality (mean = 3.0 ± 0.7), with a mean perceived fitness of 7.1 ± 1.5 on a 10-point scale. Students reported approximately 2.4 h of high-intensity and 2.2 h of light physical activity per day during the previous month.

**Table 2:** Descriptive statistics of latent constructs.

Latent construct	Item	Description	Mean	Median	S.d.	Min.	Max.
Sleep quality (SLQ)	psqi	Respondents’ sleep quality index based on the Pittsburgh Sleep Quality Index (PSQI)	16.7	17	1.9	7	21
Injury (INJ)	injury_no	Injury number	1.1	1	1.3	0	9
Lifestyle (LFS)	smoke_cig	Respondents’ number of cigarettes daily	1.2	0	3.8	0	30
	drinks_alc_am	Respondents’ number of drinks during a night out	3.2	3	4.2	0	18
	drinks_ene_am	Respondents’ number of energy drinks daily	0.9	1	0.8	0	4
Sport (SPO)	sport_no	Number of different sports	0.8	1	0.5	0	4
	sport	Respondents’ sport status	0.8	1	0.4	0	1
	sport_train	Respondents’ weekly training volume (hours)	7.5	6	5.1	0	35
	sport_comp	Respondents’ competitive sport status	1.7	2	0.6	1	3
Fitness (FIT)	health	Respondents’ health status	3.2	3	0.7	1	4
	life_qual	Respondents’ life quality status	3.0	3	0.7	1	4
	fitness	Respondents’ fitness	7.1	7	1.5	1	10
	hpa_h	High physical activity in the last 30 days: hours per day	2.4	2	1.9	0	21
	lpa_h	Light physical activity in the last 30 days: hours per day	2.2	2	1.9	0	16

“S.d.”, stands for standard deviation.

### 3.3 Empirical methodology

The empirical methodology consists of two stages. In the first stage, techniques for assessing the reliability and validity of the latent constructs used in the study were applied to ensure accuracy and appropriateness for measuring the intended constructs. The second stage involved assessing the structural research model using the partial least squares structural equation modelling (PLS-SEM) technique.

The decision to use PLS-SEM instead of covariance-based SEM (CB-SEM) was based on several reasons (60). Firstly, the objective was to predict key constructs and identify their key determinants. Secondly, the sample size was relatively small, and the data were unlikely to follow a normal distribution. Additionally, Sanchez (15) emphasises that CB-SEM assumes that an underlying accurate model generates the observed data, and the goal is to uncover this actual model based on the observed covariances. This implies that the data must conform to the model assumptions. On the contrary, PLS-SEM does not assume a specific data-generating model. Instead, it provides valuable insights into the collected data, primarily summarising the relationships between the latent variables in the model (61). Given that the research conducted in this study is exploratory and not based on well-established theories, PLS-SEM is considered an appropriate approach. Analysis was carried out using Stata 18 software.

## 4 Results

### 4.1 Measurement model

In terms of the reliability of the items and the psychometric properties of the estimated latent constructs (Table A1), all item loadings are found to be greater than or equal to 0.5 and statistically significant up to 1%. Dillon-Goldstein’s rho (DG), Cronbach’s alpha (CA), and composite reliability (CR) were considered to indicate acceptable internal consistency if ranging from 0.6 to 0.7 and good internal consistency if exceeding 0.7. Convergent validity is ensured by examining the average variance extracted (AVE) greater than 0.5 for all the constructs. The Fornell–Larcker criterion, which evaluates discriminant validity by comparing the square root of the AVE with correlations among latent variables, confirms that in every instance the square root of the AVE exceeds the highest correlation with any other construct (see Table A2). This confirms that the constructs examined in this study possess adequate discriminant validity and are not highly correlated with each other.

While most indicators demonstrated acceptable reliability and validity, we note that some constructs—particularly Lifestyle and Fitness—did not fully reach the conventional thresholds for Cronbach’s alpha, composite reliability, or AVE (see Table A1). This is not unusual in PLS-SEM applications within behavioral and health sciences, where constructs are often complex and measured with self-reported items. Cronbach’s alpha, in particular, is known to underestimate reliability in PLS-SEM, which is why Dillon–Goldstein’s rho and composite reliability are emphasized as more appropriate indicators (60). In our case, Dillon–Goldstein’s rho and CR values approached acceptable thresholds, and all loadings (except two items related to fitness) were statistically significant, supporting indicator reliability. Discriminant validity was also established through the Fornell–Larcker criterion.

Although the AVE values for certain constructs were below 0.50, convergent validity in exploratory PLS-SEM research can still be considered acceptable when strong theoretical justification exists for the constructs’ inclusion and when predictive relationships remain robust (4). Importantly, additional diagnostics confirmed the absence of collinearity or common method bias (all VIFs <3.3). For these reasons, we retained all constructs in the final model but acknowledge this as a measurement limitation that future research should address with refined scales or alternative operationalizations.

To further address concerns about common method bias (CMB) and multicollinearity, we examined full collinearity variance inflation factors (VIFs) for all constructs, following recent methodological recommendations (4, 13). All VIF values were well below the conservative threshold of 3.3, indicating that neither common method bias nor multicollinearity poses a concern in our model. This VIF-based diagnostic has been increasingly recommended as a substitute for Harman’s single-factor test, which has been criticized for its limited sensitivity and diagnostic power.

### 4.2 Structural model

Results of our structural model are presented in Table 3, Panel A. The relationship between lifestyle habits (LFS) and perceived physical fitness (FIT) was found to be negative but not statistically significant. This indicates that, although unhealthy lifestyle behaviors such as smoking, alcohol, and energy drink consumption are generally detrimental to physical fitness, this effect was not sufficiently strong or consistent in the current sample to reach statistical significance. Therefore, H1 is not supported.

**Table 3:** Hypothesis testing.

*Panel A: Overall sample*			
Hypothesis	Path	Coefficient estimate	Status
H1	LFS → FIT	−0.061	Not supported
**H2a**	**SPO → FIT**	0.243***	**Strongly supported**
H2b	LFS × SPO → FIT	−0.105**	Supported
**H3**	**LFS → SLQ**	−0.161***	**Strongly supported**
**H4**	**SLQ → FIT**	0.275***	**Strongly supported**
H5	FIT → INJ	−0.044	Not supported
**H6**	**SLQ → INJ**	−0.111***	**Strongly supported**

*Panel B: Results by gender*			
Hypothesis	Path	Coefficient estimate	
		Female	Male
H1	LFS → FIT	−0.018	−0.090
H2a	SPO → FIT	0.345***	0.223***
H2b	LFS × SPO → FIT	−0.145**	−0.073*
H3	LFS → SLQ	−0.216***	−0.168***
H4	SLQ → FIT	0.255***	0.262***
H5	FIT → INJ	−0.035	−0.062
H6	SLQ → INJ	−0.122**	−0.123***

***, **, and * denote significance levels at *p* < 0.01, *p* < 0.05, and *p* < 0.1, respectively. SLQ, Sleep quality; INJ, Injury; LFS, Lifestyle; SPO, Sport; FIT, Fitness. Bold values indicates the hypotheses that are strongly supported by our model.

Sports activity (SPO) showed a positive and statistically significant correlation with physical fitness, confirming that greater involvement in sports, through higher training volume, competitive level, or a wider variety of sports, is strongly associated with higher self-reported physical fitness. This strong support for H2a aligns with prior research emphasizing the fitness-enhancing benefits of regular and structured physical activity (33, 34).

The joint effect of lifestyle habits and sports activity (LFS × SPO) was found to have a negative and statistically significant association with fitness. This supports H2b, suggesting that the negative impact of poor lifestyle choices on physical fitness becomes more pronounced among students who are also engaged in sports. This result may imply that unhealthy lifestyle behaviors, such as smoking or excessive drinking, may counteract some of the physical benefits typically gained from sports participation.

There is a statistically significant negative association between lifestyle (LFS) and sleep quality (SLQ), confirming H3. This means that students with poorer lifestyle habits tend to report worse sleep quality, which is consistent with existing literature linking substances such as alcohol, nicotine, and energy drinks to sleep disturbances (62, 63).

Sleep quality (SLQ) was shown to have a positive and significant association on physical fitness (FIT), thereby strongly supporting H4. This suggests that students who experience better sleep quality tend to report higher levels of physical fitness, underscoring the important role of sleep in physical health and recovery (64, 65).

Surprisingly, the direct relationship between physical fitness (FIT) and injuries (INJ) was not statistically significant, indicating that better physical fitness does not necessarily correlate with a lower incidence of injuries that cause absenteeism or academic disruption. Therefore, H5 is not supported. This may reflect that injuries are influenced by other factors such as training load, sport type, or accidental circumstances rather than fitness alone.

Finally, there is a significant negative relationship between sleep quality (SLQ) and injuries (INJ), providing strong support for H6. This implies that poorer sleep quality is associated with a higher number of injuries, consistent with evidence suggesting that inadequate sleep increases the risk of physical injury due to impaired recovery, concentration, and coordination.

A separate analysis was conducted according to the students’ gender (Table 3, Panel B). Overall, the findings indicate that the core relationships observed in the full sample are largely consistent across genders, but some differences emerged in the strength of effects. For example, sports activity had a stronger positive effect on fitness among females (*β* = 0.345, *p* < 0.01) compared to males (*β* = 0.223, p < 0.01). Similarly, the negative interaction between lifestyle and sports activity on fitness was more pronounced in females (*β* = −0.145, *p* < 0.05) than in males (*β* = −0.073, *p* < 0.10). The relationships between lifestyle and sleep quality, sleep and fitness, and sleep and injury were significant and similar in both subgroups, reinforcing the robustness of the sleep-protection effect across genders.

The findings highlight the complex interrelationships between lifestyle, sleep, fitness, and injury occurrence. While sports activity and sleep quality emerge as key positive contributors to physical fitness, unhealthy lifestyle behaviors detract from both fitness and sleep quality. Moreover, sleep quality—more so than physical fitness—plays a critical role in injury prevention, reinforcing the importance of adequate rest in students’ health management. These insights may inform interventions aimed at promoting healthier lifestyle choices, improved sleep hygiene, and safer engagement in physical activity among university students.

Tables A4, A5 show the model’s predictive power and goodness-of-fit measures of the main model. The relative goodness-of-fit (GoF) index, which considers both the measurement and structural model performance, is reported to be 0.862, indicating an acceptable level of fit. Regarding SRMR, PLS-SEM traditionally focuses on predictive validity rather than overall fit, so the value of 0.092 is considered tolerated.

## 5 Discussion

The results of our structural model provide several important insights into the interactions between lifestyle habits, sleep quality, physical fitness, and injury incidence among the student population. Although unhealthy lifestyle habits were hypothesized to negatively impact physical fitness, a statistically significant relationship was not found. This suggests that while unhealthy behaviors like smoking, alcohol consumption, and energy drinks generally harm physical fitness, their impact in this sample was not strong enough to reach statistical significance. Thus, hypothesis H1 is not supported.

In contrast, sports activity proved to be a positive and significant factor for physical fitness, confirming hypothesis H2a that greater involvement in sports is associated with better self-reported physical fitness. This aligns with previous research highlighting the benefits of regular and structured physical activity.

The interaction between lifestyle habits and sports activity showed a negative and statistically significant effect on fitness, supporting hypothesis H2b. This finding suggests that poor lifestyle choices can diminish the physical benefits usually derived from sports participation. Therefore, while sports activity has a positive impact, it cannot completely neutralize the negative consequences of unhealthy behaviors.

A significant negative correlation between poor lifestyle habits and sleep quality was confirmed, consistent with hypothesis H3 and prior research linking the consumption of alcohol, nicotine, and energy drinks to sleep disturbances. Sleep quality, in turn, has a positive and significant effect on physical fitness, strongly supporting hypothesis H4 and indicating the crucial role of sleep in maintaining physical health and recovery.

Unexpectedly, the direct relationship between physical fitness and injury incidence was not statistically significant, indicating that good physical fitness does not necessarily reduce the risk of injury. Factors such as training load, type of sport, and accidental circumstances may play a more significant role.

Additionally, the significant negative correlation between sleep quality and injuries suggests that poor sleep quality increases the risk of injuries, emphasizing the importance of sleep in injury prevention.

Analysis was also performed across different genders. These subgroup findings suggest that while the protective role of sleep quality is consistent for both male and female students, the extent to which unhealthy lifestyle behaviors attenuate the benefits of sports participation may be stronger among females. This points to potentially relevant nuances when considering tailored prevention and intervention strategies.

It is also important to emphasize that this study was conducted within a specific temporal and spatial context. Lifestyle habits among students evolve over time and may differ substantially across regions. To our knowledge, this is the first study of its kind conducted in Croatia, where distinct lifestyle patterns and risk factors for injuries may exist compared to other populations. Given the limited evidence in this area, our findings highlight the need for future research exploring temporal and cross-national differences in student risk factors, for instance, by comparing populations across European universities.

These findings reveal how lifestyle habits, sleep quality, and sports activity interact to influence the physical health of the student population and can serve as a basis for designing preventive programs aimed at promoting healthier lifestyle choices, improved sleep hygiene, and safer sports participation among students.

## 6 Conclusion

This study highlights the complex relationships between lifestyle habits, sports activity, sleep quality, and physical fitness among students. Key conclusions are that, although unhealthy habits did not directly have a statistically significant impact on fitness, they show a negative effect on sleep quality, which indirectly harms fitness. Sports activity significantly improves physical fitness, but unfavorable behaviors can diminish these benefits. Sleep quality proved to be crucial for physical health, positively affecting fitness and reducing the risk of injuries, whereas fitness alone did not directly reduce injuries. These insights suggest that interventions should focus on promoting healthier habits, improving sleep, and ensuring safe sports activities for better student health.

While this study provides significant insights into the interactions between lifestyle habits, sports activity, sleep quality, and physical fitness among the student population, several limitations should be noted. First, the cross-sectional research design does not allow for inferences about causal relationships between the examined variables. Instead, results are only to be interpreted in terms of correlations and associations. Second, the reliance on self-reported data may introduce subjective biases, potentially affecting the accuracy of the results. Thirdly, the student sample may not be sufficiently representative to generalize findings to broader populations, as lifestyle habits and health outcomes are subject to cultural and social variables. Furthermore, students studying at a faculty related to health or sports science are fundamentally more committed to a healthy lifestyle. This problem could have been solved in subsequent research, for example, by involving a control group of students from other faculties. Fourth limitation concerns the operationalization of injury data. In this study, we were only able to capture injuries that led to class absence or hindered academic obligations, as these were the measures available in the survey. We did not have access to medical-attention or functional-limitation records. Future research should aim to adopt standardized injury surveillance protocols, such as those recommended by the International Olympic Committee Injury and Illness Epidemiology Consensus Group et al. (66), which would allow for more accurate and comparable estimates of injury incidence in student populations. Fifth, the lack of control over potential external factors, such as stress or diet, may have also influenced the results. Despite these limitations, the study contributes to understanding the complex interactions affecting student health. Sixth, although our cross-sectional PLS-SEM approach allowed us to examine complex interrelationships, it did not account for the positively skewed distribution and recurrent nature of student injuries. Finally, although we did not apply Harman’s single-factor test, we relied on full collinearity VIF diagnostics, which are increasingly recommended as a more rigorous approach to assess common method bias in PLS-SEM. The consistently low VIF values (<3.3) suggest that our findings are unlikely to be biased by common method variance. Also, the Lifestyle and Fitness constructs did not fully meet conventional reliability and validity thresholds. This issue is common in PLS-SEM when multidimensional constructs are measured through self-reports. Still, Dillon–Goldstein’s rho and composite reliability were close to acceptable levels, discriminant validity was established, and collinearity checks ruled out bias. Future studies should refine these measures with broader or more objective indicators.

Future research should incorporate longitudinal approaches and more diverse samples to further explore and validate these findings. They should also expand the list of observables to sedentary time or dietary intake to cover the lifestyle spectrum more fully. Future research should also consider incorporating training load indicators, such as session-rated perceived exertion (sRPE) or the acute chronic workload ratio (ACWR). These measures would allow for dose–response analyses and provide deeper mechanistic insights into the relationship between physical activity, sleep, fitness, and injury risk, thereby enhancing the explanatory power and precision of the model.

## Data Availability

The datasets presented in this article are not readily available because dataset is not allowed to be shared. Requests to access the datasets should be directed to mario.kasovic@kif.unizg.hr.
