# Supplementary material for: From habits to harm: the effects of lifestyle, sleep, and fitness on injury risk in students using a structural equation modeling approach
Source: Front Public Health. 2025 Nov 18;13:1664822. doi: 10.3389/fpubh.2025.1664822 (PMC12669233; doi:10.3389/fpubh.2025.1664822)
Supplement: Supplementary file 1 [file Supplementary_file_1.docx]

**Table A1.** Measurement model.

| **Latent construct** | **Items** | **Loading** | **CA** | **DG** | **CR** | **AVE** |
| --- | --- | --- | --- | --- | --- | --- |
| Lifestyle (LFS) | smoke_cig | 0.76*** | 0.57 | 0.74 | 0.48 | 0.48 |
|  | drinks_alc_am | 0.65*** |  |  |  |  |
|  | drinks_ene_am | 0.67*** |  |  |  |  |
| Sport (SPO) | sport_no | 0.69*** | 0.60 | 0.76 | 0.59 | 0.46 |
|  | sport | 0.74*** |  |  |  |  |
|  | sport_train | 0.66*** |  |  |  |  |
|  | sport_comp | 0.58*** |  |  |  |  |
| Fitness (FIT) | health | 0.78*** | 0.67 | 0.75 | 0.62 | 0.54 |
|  | life_qual | 0.78*** |  |  |  |  |
|  | fitness | 0.68*** |  |  |  |  |
|  | hpa_h | 0.16*** |  |  |  |  |
|  | lpa_h | 0.07*** |  |  |  |  |

Notes: (***) denotes the significance level at p<0.01. CA – Cronbach's alpha, DG – Dillon–Goldstein's rho, CR – composite reliability, AVE – average variance extracted.

**Table A2.** Fornell–Larcker criterion for assessing discriminant validity.

|  | LFS | SPO | FIT |
| --- | --- | --- | --- |
| LFS | (0.488) |  |  |
| SPO | 0.001 | (0.463) |  |
| FIT | 0.028 | 0.054 | (0.343) |

Notes: Square roots of average variance extracted (AVE), as discriminant value indicators, are shown on a diagonal line in parentheses. Abbreviations: SLQ – Sleep quality, INJ – Injury, LFS – Lifestyle, SPO – Sport, FIT – Fitness.

**Table A3.** Collinearity VIF values.

| **Latent construct** | **Items** | **VIF** |
| --- | --- | --- |
| Lifestyle (LFS) | smoke_cig | 1.083 |
|  | drinks_alc_am | 1.056 |
|  | drinks_ene_am | 1.085 |
| Sport (SPO) | sport_no | 1.001 |
|  | sport | 1.101 |
|  | sport_train | 1.113 |
|  | sport_comp | 1.113 |
| Fitness (FIT) | health | 1.338 |
|  | life_qual | 1.366 |
|  | fitness | 1.140 |
|  | hpa_h | 1.110 |
|  | lpa_h | 1.103 |

Notes: Square roots of average variance extracted (AVE), as discriminant value indicators, are shown on a diagonal line in parentheses. Abbreviations: SLQ – Sleep quality, INJ – Injury, LFS – Lifestyle, SPO – Sport, FIT – Fitness.

**Table A4.** Predictive power measures.

| **Construct** | **Adjusted** $\boldsymbol{R}^{\boldsymbol{2}}$ |
| --- | --- |
| SLQ | 0.025 |
| FIT | 0.189 |
| INJ | 0.251 |

**Table A5.** Goodness of fit measures.

| **Measure** | **Value** |
| --- | --- |
| Average $R^{2}$ | 0.209 |
| Average communality (AVE) | 0.695 |
| Absolute GoF | 0.364 |
| Relative GoF | 0.862 |
| Average redundancy | 0.147 |
| SRMR | 0.092 |
| NFI | 0.896 |

Abbreviations: AVE – Average variance extracted, GoF – Goodness of fit, SRMS – Standardized Root Mean Square Residual, NFI – Normed fit index.
